# Prevalence of Polycystic Ovarian Syndrome and Its Link to Obesity in Adolescent Girls

**DOI:** 10.7759/cureus.45405

**Published:** 2023-09-17

**Authors:** Muskan Aggarwal, Swarupa Chakole

**Affiliations:** 1 Medicine, Jawaharlal Nehru Medical College, Datta Meghe Institute of Higher Education and Research, Wardha, IND; 2 Community Medicine, Jawaharlal Nehru Medical College, Datta Meghe Institute of Higher Education and Research, Wardha, IND

**Keywords:** hyperandrogenemia, hirsutism, central adiposity, obesity, adolescent, polycystic ovarian syndrome

## Abstract

Polycystic ovarian syndrome (PCOS), also referred to as Stein-Leventhal syndrome, happens to be one of most common hormonal disorders found in females, causing large-sized ovaries with small cysts of non-ovulated oocytes in the outer medulla part of the ovary. Women suffering from PCOS often exhibit symptoms like oligomenorrhoea, elevated testosterone levels, acne, alopecia, hirsutism, sudden weight gain and many more. It can predispose a woman to developing infertility in future, and thus, difficulties in conceiving; due to the cystic changes in the ovaries, it results in anovulation and amenorrhea. The early symptoms of PCOS are being commonly observed nowadays in young women who are in their early 20s and those who are overweight or obese. The metabolic expression of PCOS increases with obesity. Obesity is a factor that is considered to contribute the most in the occurrence of various long-standing and non-transmissible illnesses apart from PCOS such as atherosclerosis, hypertension, diabetes, high blood cholesterol and even certain types of cancers. In obesity, there is an increase in the size and number of fat cells in the body. Obese and overweight young girls have a heightened likelihood of developing PCOS and its corresponding metabolic and reproductive health complications.

## Introduction and background

Obesity in both industrialized and developing nations, and in young and old, is the commonest kind of malnutrition to be seen. It stands as the sixth most important risk factor for death across the globe [1]. For an obese person, obesity also accounts for majority of other chronic and noncommunicable illnesses to develop in future [1]. Polycystic ovarian syndrome (PCOS) is now a lifestyle disorder affecting a small but significant percentage (7%) of reproductive age females [2]. The condition is complex and a diverse syndrome having manifestations throughout the adolescence, and is characterised by anovulation and androgen excess [3].

Obesity and PCOS appear to be on the rise globally. Obesity, in and of itself, is not a trait in many regions of the world. The significant prevalence of PCOS in slim individuals suggests that obesity does not seem to be an underlying factor for the condition. On the contrary, obesity compounds for a number of phenotypic characteristics, notably cardiovascular risk factors including insulin resistance and imbalance in the levels of lipids. It is also linked to an unsatisfactory outcome from infertility therapy and, in women who do conceive, a greater probability of complications during pregnancy [4]. At the time of puberty, the female body undergoes a lot of changes, specially in terms of reproductive hormones and menstrual patterns, and due to these changes, often the symptoms of PCOS are ignored; hence, it goes undiagnosed only to emerge later as a multi-system disorder causing long-term dysfunction and makes determining the cause challenging [4].

Excessive adipose tissue around the abdomen due to obesity initiates metabolic and endocrine instability, thus progressing the manifestations of PCOS. Due to the amplified androgen production in the adipose tissue, there is an increase in the testosterone concentration in the body, leading to obesity-associated hyperandrogenemia. The negative feedback of progesterone is generally inhibited by increased androgen levels in the body; this raised free testosterone concentration inside cells causes the levels of sex hormone binding globulin (SHBG) to reduce ultimately resulting in the disruption of a normal menstrual cycle [5].

A body mass index (BMI) of 25-29.9 kg/m^2^, 30-34.9 kg/m^2 ^and more than 35 kg/m^2^ in adults and the same for the age percentile above 95 for children is defined as overweight, moderately obese and severely obese, respectively; being overweight is commonly assumed to be a contributory factor in the pathogenesis of PCOS and to have a deleterious impact on its clinical manifestation. It has been suggested in a recent study conducted among teenage girls from ages 15-19 years that the rate of PCOS prevalence was 3.0 times in overweight, 6.7 times in moderately obese and 14.7 times in extremely obese girls [3].

PCOS symptoms often appear at the beginning of teenage years and many researchers believe that prepubescent obesity increases the likelihood of teenage PCOS [6,7]. Obesity is common with PCOS; however, this disease can be observed in women both normal weight and overweight. Existing evidence suggests that overweight and obesity usually mount to a severe clinical progression of PCOS, in comparison to a standard body weight. Therefore, obese women with PCOS face severe risks to their general health and quality of life.

Particularly, the rapid surge in childhood obesity has caused a rise in the frequency of the disease in adolescent girls [3,4]. The co-morbidities associated with PCOS not only manifest as anovulatory infertility but also predisposition to metabolic syndrome, type 2 diabetes mellitus, hyperinsulinemia and increased resistance to insulin [8,9].

## Review

This article is a review of published manuscripts along with existing diagnostic and clinical practices found relevant to obesity and PCOS in pubertal girls. A thorough study of the available research and pertinent studies on the subject of interest was conducted using PubMed, Science Direct, MEDLINE, Scopus, and Google Scholar indexes. There were no time or linguistic restrictions. Additional documents, synonyms, and phrase extensions were utilized to get an even more comprehensive search, and subsequently assessed keywords were used: “Rotterdam criteria for PCOS”, “hyperandrogenesim”, “ovarian follicles and volume”, “obesity and its link to PCOS”, “pubertal girls”, “young women”.

Pathophysiology

According to the Rotterdam criteria, ovaries that are polycystic (more than 12 follicles measuring 2-9 mm in diameter and/or an ovarian volume of more than 10 mL in at least one ovary) are classified as having two or three of the following characteristics: oligomenorrhea or anovulation, hyperandrogenism, and ovaries having multiple cysts [5].

It is believed that various environmental factors and genetic interactions have an impact on the aetiology of PCOS. Therefore, it is necessary to take into consideration the probability of a young girl being diagnosed with PCOS if polycystic ovaries or the syndrome is present in both the mother and the adolescent, or whenever one of the parents has obesity, diabetes mellitus, or other conditions that make the body resistant to insulin. It is crucial to spot PCOS's early symptoms throughout adolescence or even earlier. In young women with hyperandrogenism, it is important to confirm the PCOS diagnosis and rule out any other potential causes of the body producing too much androgen [3]. Adolescence is an important developmental period in which the condition develops, and its symptoms commonly appear during this period. PCOS's diagnosis in teenagers is still debatable despite the fact that the physiological and hormonal changes associated with puberty also correspond with its symptoms. Therefore, determining if the occurrence of prepubertal obesity along with its current rise has a substantial influence on the occurrence of adolescent PCOS is challenging [10,11].

According to a recent study, persistent prepubertal obesity along with severe hyperglycemia and resistance to insulin may be a precursor to the later onset of PCOS [10]. It was discovered that females who were overweight, moderately obese, or highly obese were respectively having an incremental risk in the likelihood of having PCOS. Furthermore, teenagers and younger adults suffering from PCOS had a considerably increased total incidence of obesity in comparison to those without PCOS [12]. However, it is unclear as to how much peripubertal obesity has a role to play in the development of PCOS, especially in kids and teenagers.

Although PCOS is one of the commonest hormonal disorders developing in females, still to date the aetiology of the same is unknown and of an idiopathic origin; it is believed that the variables involved in the etiopathogenesis of PCOS are abnormal ovarian steroidogenesis, neuroendocrine function, metabolism, hyperinsulinemia, insulin resistance, inflammatory factors, adipose cell function, environmental factors like food choices and lack of exercise [3,7]. In other words, it will not be wrong to say that the variability of PCOS can be described as the interplay of genetically determined ovarian condition with the environmental factors, especially dietary.

Abnormal Ovarian Steroidogenesis

In reaction to the levels of luteinizing hormone (LH) and insulin, theca cells isolated from the ovaries of women with PCOS produce an overabundance of androgens. Acute gonadotropin-releasing hormone (GnRH) agonism or human chorionic gonadotropin injection are used to evaluate ovarian steroidogenesis in vivo, and women with PCOS show that these stimuli result in enhanced 17-hydroxyprogesterone (17OHP) and androstenedione responses [3]. This "functional ovarian hyperandrogenism" (FOH) is observed in roughly 66% of women with PCOS and late adolescents and may be exacerbated by obesity-related hyperinsulinemia [13-15].

Hyperandrogenism is caused by defects in adrenal steroidogenesis (functional adrenal hyperandrogenism) in certain women with PCOS. Dehydroepiandrosterone sulphate (DHEAS) levels are high in 20%-30% of PCOS women [16]. The reactivity of the adrenal androgen (DHEA, androstenedione) may increase in obese women without PCOS, when stimulated. Thus, overactivity of enzymes and hormones of the ovarian and adrenal steroidogenic pathway along with insulin resistance, obesity and overweight play a contributory role in the progression of the disease in adolescents.

Abnormal Neuroendocrine Function

The ovarian (theca cell) androgen synthesis is stimulated by LH. In response to the changed circadian rhythm of LH secretion, young women with hyperandrogenemia and/or PCOS have an increased LH (also implicating GnRH) pulse frequency, amplitude, and ratio, also leading to increased levels of follicle-stimulating hormone (FSH). During the pubertal transition, several anomalies start to appear. The use of GnRH agonists, which are long-acting, suppress gonadotropin production and lower androgens to the levels that are usually seen in women who have undergone oophorectomy, demonstrates the significance of LH in adult PCOS hyperandrogenemia [17]. As seen in older females with and without PCOS, as well as adolescents, obesity is similarly related to decreased median LH levels and its related pulsing amplitude, with no discernible influence on the pulsatile frequency of LH. The decreased metabolic half-life of endogenous LH and decreased pituitary response to gonadotropin-releasing hormone are the two reasons that cause this to occur in adult PCOS [18].

Excessive androgen concentration and body weight (obesity) may have distinct impacts on LH secretion, with the hormone being a key contributor and a permissive factor in certain obese teenagers' enhanced ovarian androgen production [3,12,18].

An increase in testosterone levels in the body as a result of increased androgen synthesis in the adipose tissue leads to obesity-related hyperandrogenemia, which often suppresses LH production, and decreasing progesterone's negative feedback. Biochemical investigation reveals a classical presentation of elevated serum androgen concentration, LH concentration and anti-Mullerian hormone, but standard levels of FSH. Thus, the dependance of LH on ovarian hyperandrogenemia explains the pubertal manifestation of PCOS.

Insulin Resistance and Hyperinsulinemia

Resistance to insulin and its excess synthesis in the body appears to be the first sign of pubertal PCOS; young adults with PCOS have resistance to insulin and its increased levels that are higher than that seen in obese adolescents without PCOS [3,19].

Insulin resistance is generally seen among women with PCOS [19]. Despite the fact that insulin resistance emerges to be partially independent of fat, still the worsening of the resistance to insulin in PCOS is apparently the work of obesity, due to which it results in compensatory hyperinsulinemia that contributes to hyperandrogenemia in a variety of ways [20]. Insulin can also raise basal and GnRH-stimulated testosterone concentrations in women with PCOS, and experimental hyperinsulinemia can boost androgen synthesis from isolated ovarian theca cells [20]. Insulin also appears to boost adrenal androgen synthesis when triggered by the adrenocorticotropic hormone (ACTH) [21]. Hyperinsulinemia also decreases hepatic SHBG production, which increases the bioavailability of testosterone, and thus can result in unfavorable effects on granulosa cells and follicular maturation. With the help of available methods to reduce insulin levels, including weight loss, metformin, thiazolidinediones, diazoxide, somatostatin, and D-chiro-inositol, an improvement in hyperandrogenemia and menstrual function can be noted in PCOS [16].

Inflammatory Factors

The consequences of obesity-related insulin resistance and metabolic syndrome may be amplified by alterations in adipokine and inflammatory cytokine production [22]. Blood levels of adiponectin, an adipokine with insulin-sensitizing characteristics, are lower in PCOS even after regulating BMI [23], and adiponectin gene variants may be associated with PCOS [24]. Inadequate levels of adiponectin promotes androgen synthesis in the ovary because adiponectin prevents theca cells from producing steroids [25]. In women with PCOS, low adiponectin levels are believed to be associated with high insulin tolerance. Adipokine visfatin, which has been identified as a modulator of resistance towards insulin, has been discovered to be raised in PCOS and may help human theca cells respond to forskolin-stimulated 17-hydroxylase activity [26].

Elevated levels of tumour necrosis factor (TNF) when observed in obese young females can accelerate the proliferation of theca cells and steroidogenesis in the ovaries [27,28]. Inflammatory cytokines that are linked to obesity can contribute to the PCOS pathogenesis. High amounts of interleukin 6 (IL-6) may also promote adrenal steroidogenesis and contribute to hyperandrogenemia [29-31].

Genetic Factors

Many studies suggest a genetic basis of PCOS. According to the existent research, intrauterine exposure to high levels of androgen causes the foetal ovaries to increase the production of LH, and thus, the predisposition to the development of PCOS in adolescence. Endocrine and metabolic aspects of PCOS are also heritable [12,31]. An integration of genetic analysis with that targeted at resolving the complexity of atypical ovarian development of follicles could contribute to a greater understanding of the underlying pathology of PCOS, according to researchers [3].

Obesity's Impact on PCOS Reproductive Manifestations

Obesity related to PCOS is frequently diagnosed on the basis of BMI measurements; however, BMI itself is an imprecise indicator of metabolically significant adiposity. Central obesity is considered as a reflection of visceral adiposity in this condition and also associated with the severity of the disease [18].

Increased adiposity is related to increased androgen concentrations and worse menstruation disruption in women and adolescents with PCOS [4,32-35]. Obesity is a major element in the development of resistance to insulin, which has been connected to anovulation, an etiological factor of the stated syndrome [32]. Greater obesity is also linked to greater androgen concentrations in those who do not have PCOS [35-38]. Females with PCOS have difficulty conceiving and maintaining pregnancy as increased levels of androgens in the body result in anovulation leading to secondary amenorrhea. It has been stated that females with PCOS have a menstrual cycle of more than 40 days and often experience breakthrough bleeding in place of a proper menstrual bleed. This association is partly explained by obesity's detrimental impact on SHBG concentration [39], because lower SHBG levels enhance testosterone bioavailability. Obesity, on the other hand, is related to higher total testosterone levels [39], implying greater androgen synthesis.

In recent studies, qualitative anomalies in the adipose tissues of women with PCOS has been observed. The adipocytes appeared to be larger with decreased insulin sensitivity, poorer lipoprotein lipase activity and altered sensitivity to catecholamines lipolytic actions [3,13]. This is particularly essential in PCOS patients; since the illness frequently manifests in adolescence, it gives them the opportunity for early intervention in order to avoid or mitigate the seriousness (acne, acanthosis nigricans and hirsutism) of dermatological and reproductive symptoms and the long-term consequences of metabolic disorder [32].

Diet is recognized as an important environmental factor in PCOS. High-sugar and high-fat diets, as well as excessive calorie intake, can worsen insulin resistance and metabolic issues in individuals with PCOS. On the other hand, lack of physical activity can contribute to weight gain and exacerbate insulin resistance, both of which are associated with PCOS. Weight reduction, in particular, has been shown to improve hyperandrogenemia, ovulatory function, and fecundity in overweight and obese women with PCOS [40]. As an extreme example, significant weight reduction following bariatric surgery is frequently associated with PCOS improvement, often to the point where the PCOS diagnosis can no longer be confirmed [41-43].

All in all, the findings show that adiposity is a key factor in the emergence of PCOS in many individuals. As a consequence, in certain at-risk teenagers who might have remained asymptomatic, obesity is likely to contribute to the onset of clinical PCOS [44].

## Conclusions

PCOS is a complex and multifaceted condition. The critical developmental stage after which obesity-related insulin resistance and hyperandrogenemia may promote the emergence of PCOS is thought to be puberty. Hyperandrogenism and hyperinsulinemia are exaggerated in overweight women with PCOS, resulting in anovulation. Thus, obesity can exacerbate PCOS symptoms and androgen excess can lead to visceral fat increase. With weight reduction, an improvement in the symptoms of PCOS is seen.
